# Seismic insights into Earth’s core

**DOI:** 10.1038/s41467-023-41725-5

**Published:** 2023-09-27

**Authors:** Lauren Waszek, Jessica Irving, Thanh-Son Phạm, Hrvoje Tkalčić

**Affiliations:** 1 James Cook University, Physical Sciences, 1 James Cook Drive, Douglas, QLD 4811 Australia; 2 University of Bristol, School of Earth Sciences, Wills Memorial Building, Bristol, BS8 1RL UK; 3 The Australian National University, Research School of Earth Sciences, 142 Mills Road, Acton, ACT 2601 Australia

**Keywords:** Geophysics, Seismology

## Abstract

Seismological advances are presented and summarized to study the Earth’s core.

## Teaser

The inner and outer cores are the most remote layers of Earth, yet have wide-ranging impacts on its evolution. Here, we detail current structural models of the core, deliberate dynamic interpretations, and highlight limitations and potential targets for future research to address.

At the centre of the Earth, and under extreme conditions, the core is physically inaccessible and difficult to observe remotely. Its structures, dynamics, and composition influence processes throughout our planet, including plate tectonics and the geomagnetic field. More fundamentally, improved comprehension of the core’s structural properties and their dynamical origins helps shed light on Earth’s formation and ongoing evolution.

The core is an iron-nickel alloy with still-debated quantities of lighter elements, constituting approximately 15% of the Earth’s volume, and separated from the bulk of the Earth by the core-mantle boundary (CMB)^1–5^. It is partitioned into two layers: the solid inner core surrounded by the fluid outer core, a convecting molten metal alloy extending to about half the Earth’s radius. The inner core grows slowly (~1 mm/yr) as material from the outer core freezes onto its surface at the inner core boundary (ICB); this mechanism helps drive convection in the outer core, and records a history of the core’s changing environments into the fabric of the inner core. Probing the inner core’s layers therefore provides insights into Earth’s past, whilst its current processes inform regarding its future.

**Fig. 1:**
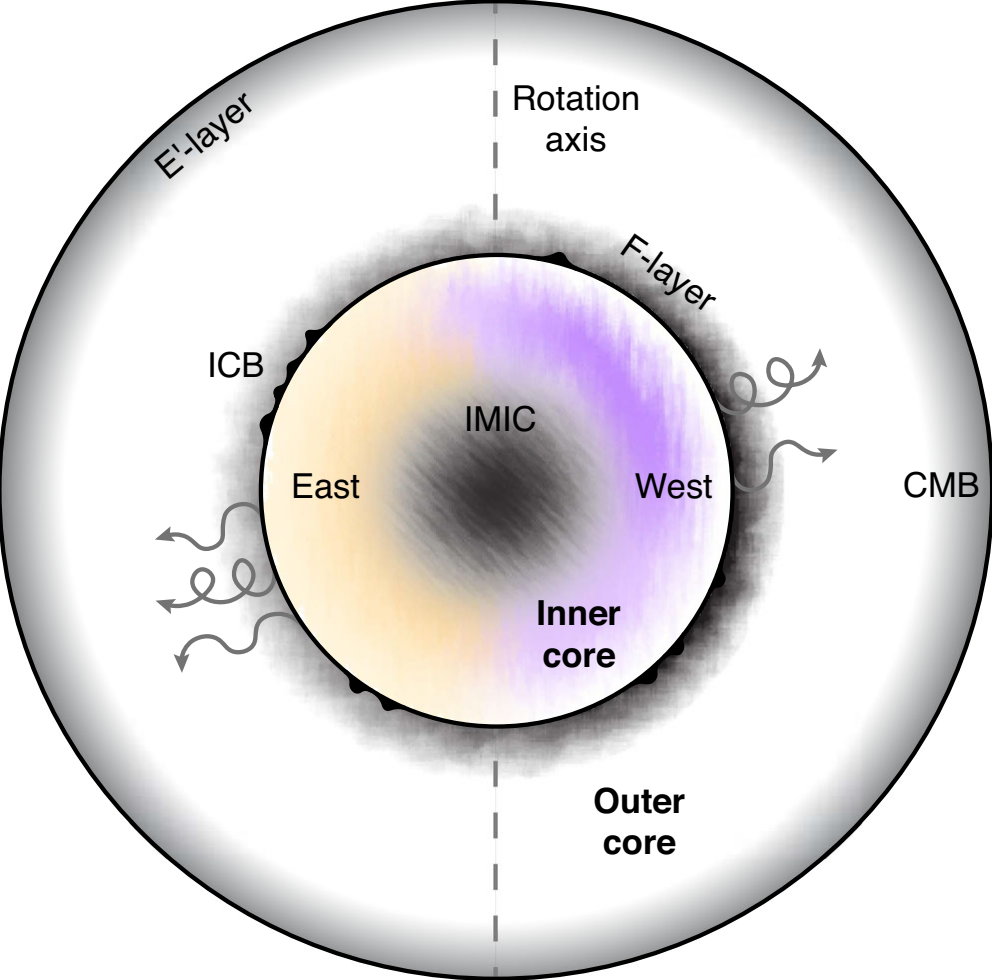
Stylised illustration of structures and processes in Earth’s core. The outer core radius is 3480 km, the inner core radius is 1220 km (to the nearest 10 km)^3^. Features denoted include: outer core stratification (the E’ and F- layers); inner core boundary topography and asymmetry of the F-layer; inner core hemispheres, anisotropy, and regional variation; and innermost inner core, distinctly different from the rest of the inner core. Arrows indicate the release of light elements upon inner core growth, colours and textures in the inner core signify the strength and nature of anisotropy, and shading in the outer core represents seismic velocity anomalies.

Heterogeneities across multiple lengthscales are observed in the core’s seismic properties^1–3^ (Fig. 1), attributed to compositional or rheological variations^5^, and linked to geodynamical processes such as convection, uneven growth, or differential rotation^1–3^. Well-established structures of the inner core include compressional wave anisotropy—a directional dependence of velocity—aligned to Earth’s rotation axis, an east-west quasi-hemispherical asymmetry, and smaller-scale features such as a further internal layer termed the “innermost inner core” (IMIC). Structurally, anisotropy is linked to intrinsic mineral anisotropy and crystal alignment, thereby informing about constituent materials^5^ and solidification or texturing processes. The low-viscosity outer core displays fewer spatial variations, with stratification in the so-called E’ and F-layers at its top and base^3^.

Our knowledge of the core relies on a combination of remote sensing seismological techniques, experimental modelling, and computational simulations. New high-quality seismic measurements provide fundamental inputs for models of dynamics and composition, the plausibility of which must be evaluated within the context of our broader understanding of constraints on the state of the core and its known physical parameters.

## Advancements in seismic techniques

Seismology’s limited observational resolution results from geographical constraints on the geometrical sampling of core-sensitive seismic waves and difficulties in isolating these small amplitude signals from noise^2^. New discoveries have employed both ever-increasing numbers of seismic stations and novel observational methods^6, 7^. These techniques deliver information beyond traditional seismic methodologies, which primarily employ the arrival times and amplitudes of direct seismic waves^1–3^ or information about whole-Earth vibrational frequencies^4^.

A significant recent development introduced the concept of the global coda-correlation wavefield^6,8–11^. Its realisation, a correlogram, is constructed by cross-correlating and stacking late hours of seismic records following large earthquakes. The long-lasting global reverberations dramatically improve spatial coverage, and the method boosts the amplitude of weak signals, manifesting as cross-correlation peaks in correlograms^6^. They have proved ideal for new observations of the core structure, including measurements of inner core shear properties^6,8^, anisotropy of the IMIC^9,10^, and stratification of the outer core^11^.

Another emerging approach uses transdimensional statistical tomography to produce 3D models of the core’s seismic properties, accompanied by uncertainty, simultaneously mapping quasi-hemispherical asymmetry, anisotropy, and regional-scale features^7,12^. Tomographic inversions for inner core structures are not new, but remain more challenging and less well-developed than mantle tomography. The most up-to-date inner core models contain large uncertainties, uneven spatial parameterisation, and theoretical errors from ambiguity in mantle structure^13^, so limitations on the resultant interpretations must be acknowledged^12^.

## Multiscale seismic structures and interpretations

We highlight here seismic structures emerging into focus within contemporary observational models, which provoke ongoing dynamical and compositional interpretations. Notable topics currently attracting discourse are inner core shear properties^3,6,8^, the IMIC^9,10,12,13^, and boundary layers^4,11,14–17^, yet inconsistencies remain in their reported properties.

### Inner core shear properties

Seismic observations confirmed the inner core as solid in the 1970s, some 30 years after mineral physics arguments proposed its solidity^1,2^. The elusiveness of inner core shear waves has restricted their ability to constrain its shear properties^3^. Recent observations of shear waves in the coda-correlation wavefield have provided improved estimates of inner core shear velocity, anisotropy, and attenuation^6,8^, proposing lower velocity and, perhaps, stronger attenuation than previous values. Updated constraints on shear velocity anisotropy near the core’s centre find the direction of the highest wavespeeds oblique to Earth’s rotation axis, in contrast to compressional velocity anisotropy^8^.

Observed shear properties of the inner core represent critical constraints for mineral physics experiments and calculations. Low shear velocity indicates softening of the inner core^5^, and the orientation of anisotropy helps ascertain candidate mineral phases of iron^1–3^. Improved geographical seismic coverage is needed for ongoing refinements to these measurements, including mapping radial and regional variations, fully calculating attenuation properties, and establishing the link to compressional velocity properties^6,8^.

### Innermost inner core (IMIC)

Until recently, the IMIC was considered a possible artefact^1^, but current work is iterating towards a consensus for its properties. A few hundred kilometres in radius, the IMIC is characterised by different anisotropy from the bulk of the inner core, with a gradual transition between the two regions. Specifically, the IMIC has stronger velocity anisotropy, and a different direction of slowest wavespeed^9,10,13^. Although it has been proposed that the IMIC displays hemispherical asymmetry^1^, recent work argues that it may instead be shifted off-centre^12,13^. However, these two analyses define the IMIC with different criteria, using the polar-equatorial difference in wavespeed^13^ versus the direction of slowest wavespeed^12^, and subsequently reach opposing conclusions for its position.

Dynamically, the IMIC may mark a gradual transition in the growth processes of the inner core, observed as a change in the crystalline structure of iron^3,10^; the relic of two-stage formation, also detected in paleomagnetic data^18^; or the onset or cessation of external mechanisms creating texturing^13^. We anticipate ongoing improvements to the current IMIC models will provide the underpinning measurements to orient the next generation of core evolution models.

### Inner core boundary (ICB)

We have considerably more data from the uppermost inner core than for deeper layers due to greater volumetric coverage and comparatively straightforward techniques to isolate the relevant signals^1,2^. The upper inner core demonstrates a distinct east-west dichotomy in its seismic velocity, the quasi-western hemisphere being slower for compressional waves by about 1%. Anisotropy is negligible in the outermost layers^1–3^, but increases considerably ~50 km beneath the ICB. It is thought to be confined to the western hemisphere^12^, although the current spatial coverage is far from complete. The ICB itself sustains localised topography of a few kilometres^2,14^.

As the locus of inner core growth, the ICB region provides insight into current dynamical processes of the inner core and its interaction with the outer core^1–4^. Its features are ascribed to various dynamical mechanisms recorded in the inner core structure as it freezes^3^. For example, asymmetric heat flow could generate hemispherical differences in the inner core, with localised enhanced flux creating dynamical topography on the ICB and compositional heterogeneity in the F-layer^1–4,14^. Temporal variation of ICB topography and the uppermost IC may also arise from the differential rotation of the inner core with respect to the mantle^19,20^. This is difficult to detect seismically, hence there is currently no consensus on rotation and/or oscillation rate^1–3,14,19,20^.

### Outer core layering

Layering may exist at both boundaries of the outer core. The ~100 km thick F-layer at its base exhibits a decreasing seismic velocity gradient^4,11^, and lateral variability^15^. In the uppermost outer core, the E’-layer displays a lower velocity anomaly relative to the well-mixed bulk of the outer core^3,4,11^, although studies disagree on its thickness^16,17^. Fluctuations in geomagnetic field strength and length-of-day measurements support the presence of stratification in the outermost outer core^21^. These data further indicate additional small-amplitude lateral variations in temperature and composition, but which are seismically undetectable^3^. Linking seismic models of the E’-layer to specific compositions is also not straightforward^5^.

The existence of the F-layer is attributed to inner core growth mechanisms causing chemical stratification; for example, a partially-frozen slush snowing onto the ICB^3^. Localised melting or freezing of the ICB could create regional enrichment of iron or light elements. There are multiple options for generating a compositionally stratified E’-layer: remnants of core formation, interaction at the CMB, or light elements released upon inner core growth^3^. At the CMB, heat is extracted unevenly by the silicate mantle; seismic investigations of this dynamically important interface are an active research area^22^.

## Outlook

As we face an unprecedented expansion of data sensitive to the deep Earth, seismic studies of the core are at a pivotal stage. We anticipate that the recent discoveries discussed here will precipitate further advancements in knowledge of core structures and dynamics. Potential near-future targets for core seismology include inner core shear wave attenuation, anisotropy, and tomography, high-resolution observations of boundary and transition layers, and improved tracking of temporal changes. Performing many of these analyses will be challenging, with several likely beyond the scope of present seismic detection techniques. Continual improvements in observational infrastructure and methodologies will therefore remain integral to the development of our understanding of the core. Coda-correlation^6^ and machine learning algorithms^23^ are emerging as valuable tools in studies of Earth’s inner layers, for seismic measurements, and their integration with outputs from other disciplines^24^. We anticipate that such data driven approaches will become prevalent for investigations into the core in coming years. Overall, interdisciplinary and multidisciplinary collaborations will be essential as we move forward in our attempts to understand Earth’s enigmatic core.
